# Tag7-Mts1 Complex Induces Lymphocytes Migration via CCR5 and CXCR3 Receptors

**Published:** 2018

**Authors:** T. N. Sharapova, E. A. Romanova, L. P. Sashchenko, D. V. Yashin

**Affiliations:** Institute of Gene Biology of the Russian Academy of Sciences, Vavilova Str., 34/5, Moscow, 119334 , Russia

**Keywords:** Chemotaxis, chemokine, chemoreceptor, Tag7-Mts1 complex

## Abstract

The discovery of new chemokines that induce the migration of lymphocytes to the infection site is important for the targeted search for therapeutic agents in immunotherapy. We recently showed that Tag7 (PGLYRP1), an innate immunity protein, forms a stable complex with the Ca^2+^ -binding protein Mts1 (S100A4), which is able to induce lymphocyte movement, although the individual Tag7 and Mts1 do not have this activity. The purpose of this study is to identify receptors that induce the migration of lymphocytes along the concentration gradient of the Tag7-Mts1 complex, and the components of this complex capable of interacting with these receptors. The study investigated the migration of human PBMC under the action of the Tag7-Mts1complex. PBMC of healthy donors were isolated using a standard Ficoll-Hypaque gradient centrifugation procedure. It has been established that the movement of PBMC along the concentration gradient of the Tag7-Mts1 complex is induced by the classical chemotactic receptors CCR5 and CXCR3. It has been shown that only Mts1 is able to bind to the extracellular domain of CCR5, however, this binding is not enough to induce cell movement. A comparative analysis of the primary and 3D structures of the three proteins revealed the homology of the amino acid sequence fragments of the Tag7-Mts1 protein complex with different sites of the CCR5 receptor ligand - MIP1α protein. In conclusion, it should be noted that the Tag7-Mts1 complex can be considered as a new ligand of the classical chemotactic receptors CCR5 and CXCR3.

## INTRODUCTION

At least two stages are required for the development of the immune response: activation of effector lymphocytes capable of killing foreign cells, and their delivery to the affected area. Therefore, in order to understand the processes of immune protection, one should understand both cytotoxic and chemotactic mechanisms [1]. The search for new stimulators of cytotoxicity and chemotoxicity is also important.

Cytokines that cause lymphocyte migration are called chemokines. One of the peculiarities of chemokines structure is characteristic disulfide bonds. Depending on the relative position of the first two N-terminal cysteine residues, chemokines are divided into four classes (CC, C, CXC, CX_3_C) [2]. Induction of chemotaxis occurs through interaction with specific chemotactic receptors. These receptors belong to a large group of transmembrane G-protein-coupled receptors [3]. The interaction of the chemokine with the receptor causes the dissociation of the β-, γ-subunits of the G-protein, which leads to the activation of the protein kinase cascade and an increase in the concentration of Ca^2+^ ions [4, 5].

The second structural feature of chemokines is their small molecular weight (from 8–10 kDa) [6]; however, there are stimulators of lymphocytes migration with both higher and lower molecular weight [7]. Recently, we have shown that the migration of lymphocytes can be caused by a complex of two proteins: Tag7 and Mts1 [8].

Mts1 (S100A4) belongs to the family of Ca^2+^-binding proteins. It is known to be involved in the process of tumor cells metastasis [9-12]. At the same time, its gene is actively expressed in cells of the immune system involved in antitumor activity. Previously we have demonstrated that Mts1 on the surface of CD4^+^ lymphocytes is involved in the recognition of HLA-negative tumor cells and promotes their lysis [13].

Tag7 protein (PGLYRP1), whose gene was discovered at our institute, is a protein of the innate immunity system that participates both in antibacterial and antitumor activity [14-16]. Like cytokines, Tag7 can activate lymphocyte cytotoxicity. In combination with the main heat shock protein, Tag7 has a cytotoxic effect on TNFR1-bearing tumor cells and inhibits tumor growth [17, 18]. It can interact with the Mts1 protein with the formation of a stable chemoattractant complex, causing the migration of lymphocytes. Taken separately, neither Mts1 nor Tag7 possess such activity [8]. Therefore, it is interesting to find out why chemotactic activity appears only after the formation of the complex.

The purpose of this study is to identify receptors that induce the migration of cells along the concentration gradient of Tag7-Mts1 and the protein of this two-component complex capable of interacting with these receptors.

## EXPERIMENTAL

**Proteins**

Recombinant proteins Mts1 (S100A4) and Tag7 (PGLYRP1) were expressed in *Escherichia coli *M15 strain [4] (Qiagen, USA) carrying pQE-30 plasmid (Qiagen, USA). cDNAs of Tag7 or Mts1 protein were previously cloned into pQE-30 plasmid. Mts1 was purified on Ni-NTA-agarose (Qiagen, USA) according to the manufacturer’s protocol. Tag7 was isolated and purified as described in [19].

Comparison of the primary and spatial structures of proteins was performed using https://blast.ncbi.nlm.nih.gov/ and https://ncbi.nlm.nih.gov/database.

**Cell cultures**

We used peripheral blood mononuclear cells (PBMCs) obtained from the leukomass of healthy donors by sequential Ficoll-Hypaque gradient centrifugation (GE Healthcare, Sweden) as described in [20].

**Flow cytofluorometry**

Cells were fixed in 4% formaldehyde (Sigma) and incubated with antibodies to CCR5 and CXCR3 (Abcam, United Kingdom) overnight, and then with anti-rabbit IgG-PE (Beckman coulter, USA) in the dark at 40 °C for 2 hours. At least 104 cells were analyzed in each sample. The measurements were performed on a Cytomics FC 500 MPL flow cytometer (Beckman coulter, USA), data were processed in EXPO32 software (Applied Cytometry Systems, Sheffield, UK).

**Analysis of chemotactic activity**

A Boyden chamber (Costar Corning Inc., USA) was used to measure the chemotactic activity. 200×10^3^ PBMC cells were added to its upper part and a chemoattractant at a concentration of 10^–9^ M in RPMI 1640 medium (Gibco, USA) was added to the lower part. MTT test (Sigma, United States) was used to measure the number of cells that passed through the membrane after 1.5 h. In the case of preincubation, antibodies (at a dilution of 1:1000) or proteins (Tag7, Mts1 at a concentration of 10^-8^ M) were added to PBMC and incubated for 1 hour at 37 °C, 5% CO_2_, and then washed twice with the medium. Unless stated otherwise, all diagrams are based on at least three independent experiments. Bilateral ANOVA was used for statistical processing.

**Chemoreceptor detection**

PBMC cells (~ 250 mln) were suspended in 1.5 ml of solubilization buffer: 50 mM Tris, pH 7.5 with PMSF (Sigma, USA) (1 mM) and a protease inhibitor cocktail (Calbiochem, Germany) at a concentration specified by the manufacturer, and Triton X-100 detergent (Sigma, USA) (1% by volume). After incubation for 30 minutes on ice on a shaker, the resulting suspension was diluted 10 times by adding solubilizing buffer free from detergents, and centrifuged at 185,000 *g* (Beckman L7 Ultracentrifuge, USA) for 1 h at 4 °C. The supernatant was collected and applied to a Br-CN-Sepharose column with conjugated Mts1. Bound proteins were separated using 12% SDS-PAGE, transferred to a nitrocellulose membrane and detected by Western blot with specific antibodies to CCR5 and CXCR3 (1:1000) and secondary anti-rabbit antibodies (1:10,000), conjugated with horseradish peroxidase, and stained with the ECL Plus kit (Amersham, UK) according to the manufacturer’s recommendations.

## RESULTS

**CCR5 and CXCR3 chemotactic receptors induce the movement of lymphocytes along the concentration gradient of the Tag7–Mts1 complex**

At the first stage of the study, we identified the receptors involved in the transmission of the chemotactic signal from the new chemokine described by us, the Tag7–Mts1 complex. Earlier, we had demonstrated that this complex can direct the movement of T-lymphocytes and NK-cells [8]. Therefore, we evaluated the presence of chemotactic receptors CCR5 and CXCR3 on PBMCs, which are most densely present on the surface of T-lymphocytes and NK cells.

**Fig. 1:**
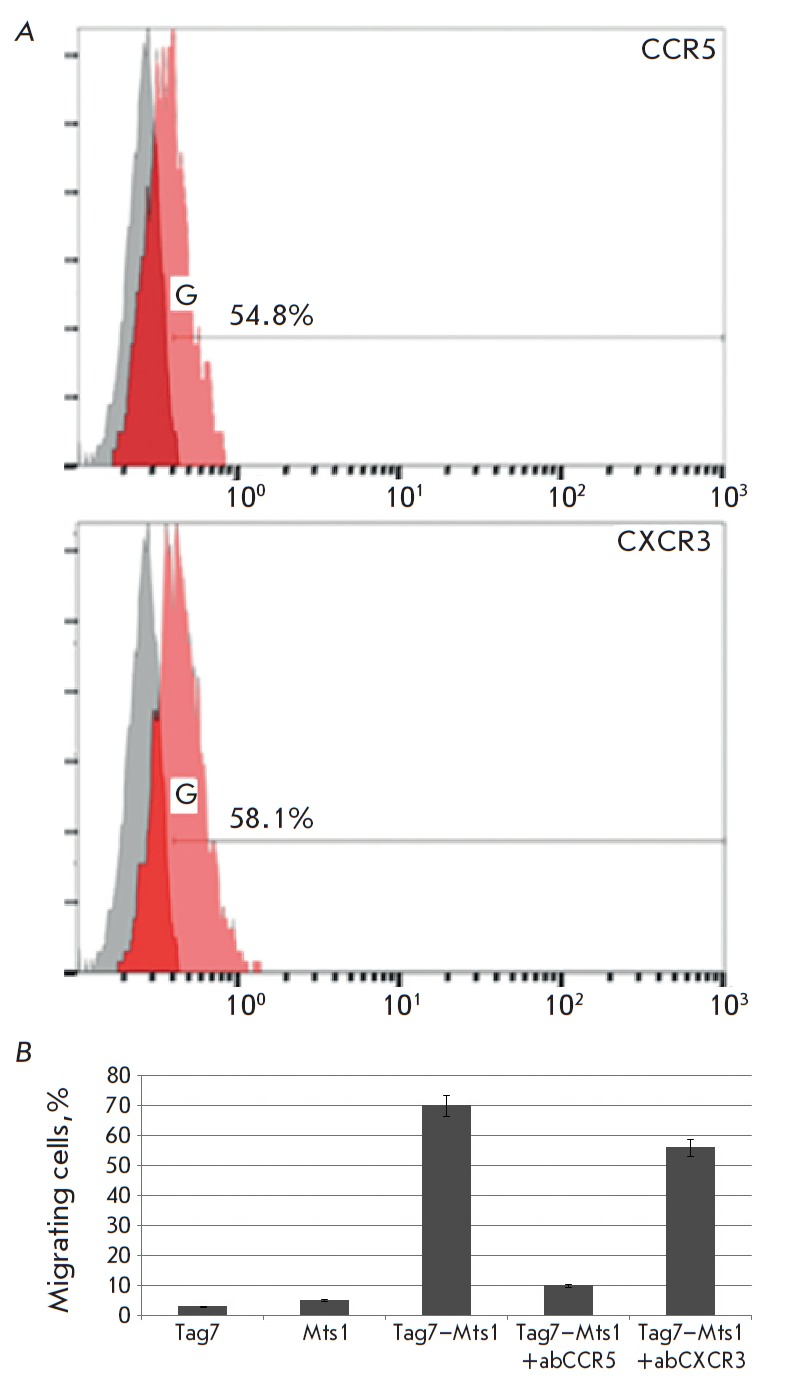
The chemotaxis of PBMC under the action of Tag7-Mts1 is achieved through interaction with CCR5 and CXCR3 receptors. *(A) *Expression of CCR5 and CXCR3 on the surface of mononuclear cells. The number of events is plotted on the abscissa axis, and the average fluorescence intensity is plotted on the ordinate axis. Gray peak – isotypic control by secondary antibodies. *(B) *Antibodies to CCR5 and CXCR3 receptors block the chemotactic activity of PBMCs

Using flow cytofluorometry and highly specific antibodies, we showed that the studied PBMC populations contain 54.8% of the cells that carry CCR5 receptor on their surface and that the cells expressing CXCR3 constitute 58.1% of the total PBMC population: i.e., both receptors are present on PBMCs (*Fig. 1A*).

We further examined whether these receptors are involved in the induction of lymphocyte migration along the concentration gradient of the Tag7–Mts1 complex. For this purpose, PBMCs were incubated with antibodies to CCR5 or CXCR3 and the movement of these cells under the action of the Tag7–Mts1 complex was investigated (*Fig. 1B*). Unlike Tag7 and Mts1 proteins separately, the Tag7–Mts1 complex causes the movement of PBMCs. Preincubation with CCR5 antibodies almost completely abolishes chemotaxis. However, CXCR3 antibodies reduced the migration of PBMCs by no more than 20%. Therefore, both studied receptors can induce cell movement along the concentration gradient of the Tag7–Mts1 complex but they display different affinity for this complex. The stronger inhibition of cell movement by antibodies to CCR5 suggests that the spatial structure of the functional regions of the Tag7–Mts1 complex involved in interaction with CCR5 is more similar to the spatial structure of the CC-chemokines regions, ligands of the CCR5 receptor which are responsible for interaction in the complex.

**Mts1 can bind to chemotactic receptors**

**Fig. 2:**
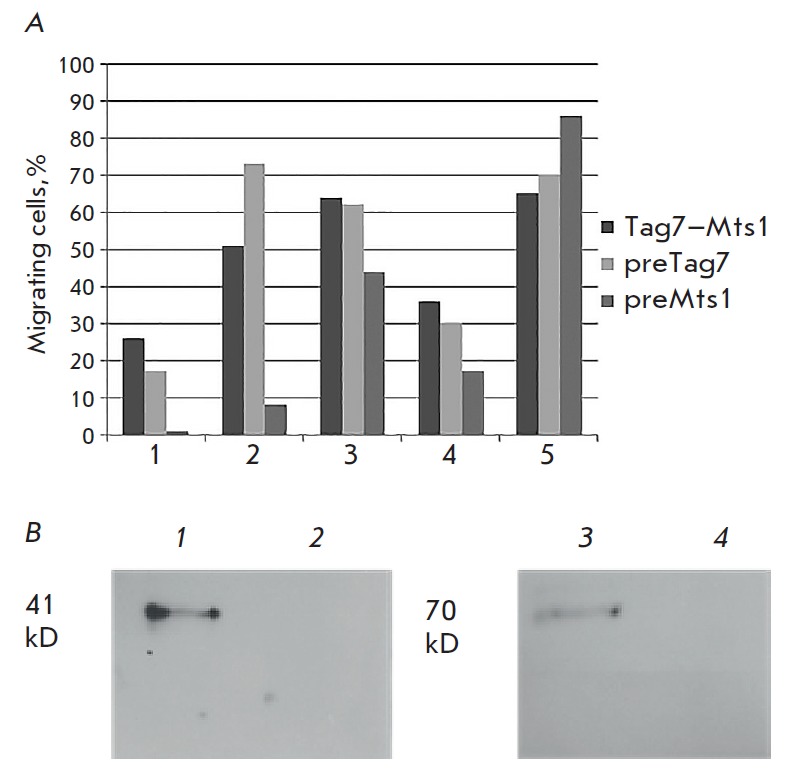
Mts1 can bind to CCR5 and inhibit chemotaxis activity. *(A) *Mts1 is able to block the PBMC chemotaxis. The abscissa axis presents the results of chemotaxis from 5 different donors. *(B) *Mts1 binds to CCR5 and CXCR3 receptors. The proteins (*1, 3*) interacting with Mts1 and unbound material (*2, 4*) were stained with specific antibodies to CCR5 (*1 *and *2*) and CXCR3 (*3 *and *4*)

Next, we determined which of the proteins of the two-component complex can interact with the receptors. We preincubated the PBMC with Tag7 or Mts1 and examined the migration of such cells under the action of the Tag7–Mts1 complex. The results of five independent experiments without averaging are presented on *Fig. 2A*. In four cases preincubation with Tag7 has virtually no effect on cell motility, whereas preincubation with Mts1 dramatically reduces the movement of PBMCs. The observed abnormalities may depend on the immune status of the donor. The similarity of observed effects in four cases suggests that Mts1 can bind to the receptor.

To test this assumption, we studied the possibility of binding of CCR5 and CXCR3 to Mts1 using affinity chromatography. Solubilized PBMC membrane proteins were applied to a column with Mts1 immobilized on Br- CN-sepharose, and the specifically bound material was analyzed using 12% SDS-PAGE followed by Western blot (*Fig. 2B*). Antibodies to CCR5 revealed a 41 kDa protein, and antibodies to CXCR3, a 70 kDa protein corresponding in molecular weight to these receptors. We can see weaker binding of CXCR3 to Mts1, which confirms the assumption of higher affinity of the chemoattractant Tag7–Mts1 complex to the CCR5 receptor.

Thus, Mts1 can bind to the CCR5 receptor, but this is not enough to induce cell movement. However, by interacting with CCR5, it prevents the binding of a two-component chemoattractant with it and inhibits the movement of cells along the concentration gradient of the Tag7–Mts1 complex.

**The primary and spatial structures of Tag7 and Mts1 fragments have partial homology with the structures of MIP1α fragments**

As already mentioned, none of the proteins in the Tag7–Mts1 complex has a standard chemokine structure referred to as a “Greek key.” Therefore, we compared the primary and spatial structures of Mts1 and Tag7 proteins and MIP1α, the known functional ligand of the CCR5 receptor.

**Fig. 3:**
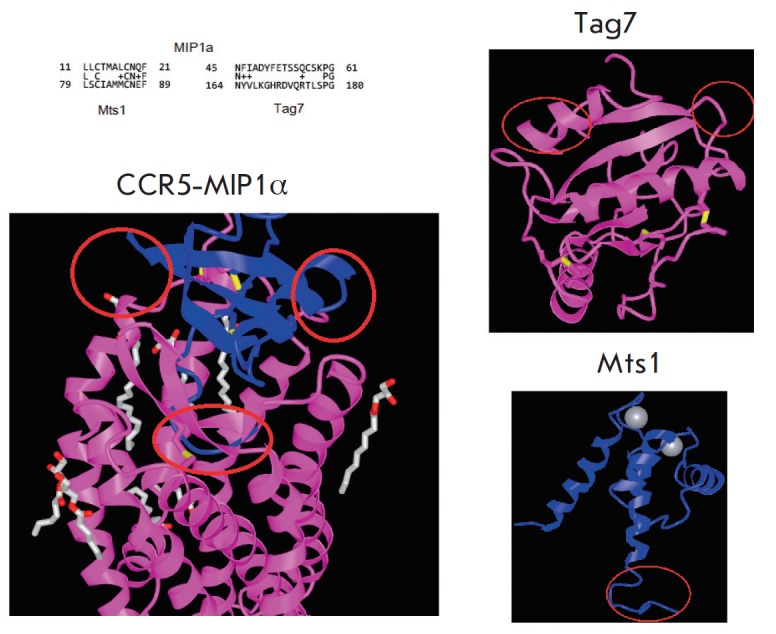
Homologous amino acid sequences and 3D structures of the Mts1, Tag7 and MIP1α proteins. In the upper left corner there is a superposition of homologous fragments of the amino acid sequences of the proteins MIP1α (above), Mts1 and Tag7 (below). On 3D models of the MIP1a complex (blue, left) with CCR5 (pink, left), and Tag7 proteins (pink, right) and Mts1 (blue, right) red areas show homology sequences of amino acid sequences

A comparative analysis of the amino acid sequences of the three proteins revealed the homology of the Mts1 and Tag7 molecules fragments with some regions of MIP1α. The result of comparing fragments of amino acid sequences is presented in *Fig. 3* (top left). In the C-terminal part, Mts1 has an 11-membered fragment (amino acid residues 79–89), 65% homologous to an 11-membered N-terminal fragment of MIP1α (amino acid residues 11–21). Tag7 has a 17-membered fragment (amino acid residues 164–180) in the central part of the molecule, which is homologous to the MIP1α fragment (amino acid residues 45–61), also located in the middle of the polypeptide chain.

*Figure 3* shows the spatial structures of the MIP1α complex with CCR5 [21] and the spatial structures of Tag7 [19] and Mts1 [22]; the coordinates of the spatial structures in PDB ID: 5UIW, 1YCK, 3C1V, respectively. The comparison of the spatial structures of the Mts1 and Tag7 proteins with the structure of MIP1α make it obvious that the C-terminal region of Mts1 (amino acid residues 79–89) is an α-helix protruding from the central globular part of the molecule. In the chemokine MIP1α, the N-terminal region (amino acid residues 11–21) also protrudes far from the central part of the molecule. Both sites have five hydrophobic amino acids. Tag7 fragments (amino acid residues 164–180) and MIP1α (amino acid residues 45–61) are β-sheets located on the surface of the molecules in both proteins. Homologous amino acids (residues 164–166 and 179–180) are located in the region that is involved in direct interaction with the CCR5 receptor in MIP1α (residues 45–47 and 60–61).

None of the proteins in the Tag7–Mts1 complex possesses the spatial structure of a chemokine, however, both Mts1 and Tag7 contain regions homologous in their amino acid and spatial structures to the MIP1α chemokine sites important for activation of the CCR5 receptor. It may be the reason why Tag7 and Mts1 taken individually do not possess chemoattractant activity, and only the stable two-component complex of these proteins can initiate the migration of lymphocytes.

## DISCUSSION

The presented data allow us to make two conclusions. CCR5 and CXCR3 chemotactic receptors are involved in the induction of PBMCs migration along the concentration gradient of the Tag7–Mts1 complex. One of the components of this complex, Mts1 protein, can bind to both receptors.

Different binding specificities of the Tag7–Mts1 complex with these receptors should be noted. The studied complex rather weakly interacts with the CXCR3 receptor: no more than 20% of CXCR3-containing PBMCs migrate along the Tag7–Mts1 concentration gradient. At the same time, almost all populations of PBMC carrying CCR5 can move under the influence of this chemoattractant.

CCR5 is present, as a rule, on memory cells, macrophages and dendritic cells. Recently, it has been shown to be present on the cell surface of NK cell subpopulations [23]. Based on a set of cells carrying CCR5, it can be assumed that the Tag7–Mts1 complex can attract cells of the immune system mainly in the early stages of the immune response.

We have demonstrated that preincubation of cells with Tag7 protein does not inhibit cell migration under the action of the Tag7–Mts1 complex. The interaction between Tag7 and the chemotactic receptor is probably much weaker than that of the Tag7–Mts1 complex. Tag7 also does not contain a hydrophobic fragment in the polypeptide chain capable of binding to the transmembrane active center of the receptor.

In contrast, Mts1 can bind to CCR5 receptor and inhibit the movement of PBMCs, although no similarities are found in the amino acid and spatial structures of the central region of the Mts1 and MIP1α molecules. The mechanism of such binding requires further study [19, 21, 22].

We have recently obtained similar results in a study of interaction of the Tag7–Hsp70 cytotoxic complex with the receptor of the well-known TNFα cytokine, TNFR1. Tag7 bound to TNFR1 and inhibited the cytotoxic effect of TNFα [24] but did not have the homology of the primary and three-dimensional structures with TNFα.

A detailed study of the mechanism of interaction of CCR5 with the ligand allowed us to propose a hypothetical pattern of contacts between this receptor and ligands [25]. According to this scheme, the interaction of the chemokine receptor with the ligand is a two-step process. In the first stage, the central part of the chemokine molecule interacts with the receptor binding center, located on the extracellular domain. Then, the interaction of the N-terminal of the chemokine with the second binding site located in the bundle of transmembrane helices is required for the activation of the receptor.

Notably, Mts1 itself cannot induce cell migration, although it has a hydrophobic fragment (amino acid residues 79–89) homologous to the MIP1α fragment (amino acid residues 11–21), which induces a change in the conformation of the receptor. Considering the differences in the spatial structure of Mts1 and in the structure of a classical chemokine, it can be assumed that after binding to the extracellular domain in the first stage of the interaction of Mts1 with the CCR5 receptor, the C-terminal fragment of Mts1 cannot penetrate into the cell membrane [22]. Interaction with Tag7 may change the conformation of Mts1, providing access of the C-terminal region to the active center in the transmembrane bundle. Such a hypothetical scheme can explain why only the Tag7–Mts1 complex can cause migration of PBMCs.

Apparently, the two-stage interaction of ligands with receptors is a common property of receptors of different nature. First, the ligand is fixed on the surface of the receptor, then it is activated. Earlier, we studied the interaction of the Tag7–Hsp70 two-component complex with the TNFR1 receptor and identified the functional activity of each protein. We have demonstrated that Tag7 can bind to TNFR1 but is not capable of causing aggregation of its cytoplasmic domains, which is necessary for the induction of cytolysis. Hsp70, which can aggregate in solution, binds to Tag7 and trimerizes the receptor.

It is possible that Mts1 can bind to other receptors on the surface of T-lymphocytes and NK cells and, in combination with Tag7, induce the migration of these cells. However, this issue requires further study.

## CONCLUSION

In conclusion, it should be noted that as a result of the studies performed, the chemotactic complex Tag7– Mts1 can be considered a new ligand of the chemotactic receptors CCR5 and CXCR3, which are present on the cells of the immune system. Although none of the proteins of this ligand has the structural motive of a classic chemokine, Tag7–Mts1 can induce the migration of PBMCs with the involvement of classical chemokine receptors and shows greater affinity for CCR5. It has also been shown that Mts1, one of the proteins of the two-component complex, can bind to the extracellular domain of CCR5; however, additional interaction of Tag7 with its extracellular region is required for receptor activation. Understanding the processes underlying the interaction of a nonclassical chemokine with a classical chemotactic receptor will help understand the mechanisms of migration of immune system cells to the affected area and the search for new chemokines.

## Abbreviations

PBMC: peripheral blood mononuclear cells
HLA: human lymphocyte antigen
CCR5 and CXCR3: chemoreceptors
TNFR1: tumor necrosis factor receptor
TNF?: tumor necrosis factor
Tag7 (PGLYRP1): an innate immunity protein
Mts1 (S100A4): Ca2+ -binding protein
MIP1?: macrophage inflammatory protein, chemokine
NK: natural killers
PMSF: p-phenylmethylsulfonyl fluoride
